# Depression and smoking characteristics among HIV-positive smokers in Russia: A cross-sectional study

**DOI:** 10.1371/journal.pone.0189207

**Published:** 2018-02-06

**Authors:** Karen E. Lasser, Karsten Lunze, Debbie M. Cheng, Elena Blokhina, Alexander Y. Walley, Hilary A. Tindle, Emily Quinn, Natalia Gnatienko, Evgeny Krupitsky, Jeffrey H. Samet

**Affiliations:** 1 Department of Medicine, Section of General Internal Medicine, Boston University Schools of Medicine and Public Health/Boston Medical Center, Boston, Massachusetts, United States of America; 2 Department of Medicine, Section of General Internal Medicine, Clinical Addiction Research and Education Unit, Boston University School of Medicine/Boston Medical Center, Boston, Massachusetts, United States of America; 3 Department of Biostatistics, Boston University School of Public Health, Boston, Massachusetts, United States of America; 4 First St. Petersburg Pavlov State Medical University, St. Petersburg, Russian Federation; 5 The Vanderbilt Center for Tobacco, Addiction, and Lifestyle, Vanderbilt University School of Medicine, Nashville, Tennessee, United States of America; 6 Data Coordinating Center, Boston University School of Public Health, Boston, Massachusetts, United States of America; 7 Department of Medicine, Section of General Internal Medicine, Clinical Addiction and Research Education Unit, Boston Medical Center, Boston, Massachusetts, United States of America; 8 St. Petersburg Bekhterev Research Psychoneurological Institute, St. Petersburg, Russian Federation; 9 Department of Community Health Sciences, Boston University School of Public Health, Boston, Massachusetts, United States of America; Harvard Medical School, UNITED STATES

## Abstract

**Introduction:**

Globally, persons with HIV infection, depression and substance use disorders have a higher smoking prevalence and smoke more heavily than other populations. These associations have not been explored among Russian smokers with HIV infection and substance use disorders. The purpose of this study was to examine the relationship between the presence of depressive symptoms and smoking outcomes in an HIV-positive cohort of Russian smokers with a history of substance use disorders (alcohol and/or drug use disorders).

**Methods:**

We performed a cross-sectional secondary data analysis of a cohort of HIV-positive regular smokers with a history of substance use disorders recruited in St. Petersburg, Russia in 2012–2015. The primary outcome was heavy smoking, defined as smoking > 20 cigarettes per day. Nicotine dependence (moderate-very high) was a secondary outcome. The main independent variable was a high level of depressive symptoms in the past 7 days (defined as CES-D > = 24). We used multivariable logistic regression to examine associations between depressive symptoms and the outcomes, controlling for age, sex, education, income, running out of money for housing/food, injection drug use, and alcohol use measured by the AUDIT.

**Results:**

Among 309 regular smokers, 79 participants (25.6%) had high levels of depressive symptoms, and 65 participants (21.0%) were heavy smokers. High levels of depressive symptoms were not significantly associated with heavy smoking (adjusted odds ratio [aOR] 1.50, 95% CI 0.78–2.89) or with moderate-very high levels of nicotine dependence (aOR 1.35, 95% CI 0.75–2.41).

**Conclusions:**

This study did not detect an association between depressive symptoms and smoking outcomes among HIV-positive regular smokers in Russia.

## Introduction

The Russian Federation (Russia) is a country with among the highest proportion of its population smoking: 12.3% of women and 38.2% of men are daily smokers [1]. Russia also has among the highest per capita cigarette consumption globally [1,2]. This tobacco burden is a significant contributor to low life expectancy; Russia has the third lowest life expectancy at birth among Organisation for Economic Co-operation and Development (OECD) countries, at 70.7 years [3]. Quit ratios (i.e., the percentage of persons who ever smoked daily who no longer smoke) in Russia are low, at less than 20% [4]. In order to improve health outcomes, smoking cessation interventions in Russia need to target populations at highest risk of smoking and who may have the most difficulty quitting due to high levels of nicotine dependence and/or the heaviest smoking. Such populations include persons with HIV infection, [5] depression [6,7], and/or substance use disorders [8].

As of 2016, approximately 1 million people live with HIV infection in Russia [9,10]. While there is a paucity of data regarding smoking prevalence among HIV-positive Russians, a recent study [11] showed that 60% of HIV-infected Russian women reported cigarette smoking in the past month. Nearly half of recent smokers were moderate or heavy smokers. Smoking among persons living with HIV/AIDS is associated with increased morbidity and mortality [12]. In addition to causing increased cardiovascular and cancer-related mortality, smoking impairs efficacy of some antiretroviral medications [13], and may increase susceptibility to opportunistic infections [14]. Kaleta et al recently evaluated patterns of nicotine dependence among Russian adults [15]. However, that study did not analyze data on HIV infection, substance use or depression. A second study in nine former Soviet republics, now independent states, showed that high levels of psychological distress have a significant association with higher levels of nicotine dependence [16]. While prior studies have documented an association between high levels of nicotine dependence and depressive symptoms among HIV-positive patients in France [17], and an association between depression and smoking among HIV-positive patients in the United States [18,19], we are unaware of studies that have examined the association between depression and smoking among HIV-positive smokers in Russia. Hypothesizing an association between depression and smoking is reasonable. According to Munafo and Araya [20], depression may cause people to smoke as a form of self-medication of depressive symptoms. Alternatively, smoking may produce an increased risk of depression via changes in neurotransmitter pathways following chronic exposure to tobacco smoke [20].

Our aim was to examine the relationship between depressive symptoms and heavy smoking in an HIV-positive cohort of Russians with a history of substance use disorders (alcohol and/or drug use disorders). We hypothesized that high levels of depressive symptoms in the past 7 days, as measured by the Center for Epidemiologic Studies-Depression Scale (CES-D) [21,22], would be associated with a higher odds of reporting heavy smoking, as compared to those without high levels of depressive symptoms. We also hypothesized that high levels of depressive symptoms would be associated with high levels of nicotine dependence, as measured by the Fagerström Test for Nicotine Dependence [23,24].

## Methods

### Study design and data sources

We performed a cross-sectional secondary analysis of data from the Russia ARCH (Alcohol Research Collaboration on HIV/AIDS) cohort recruited in St. Petersburg, Russia. Russia ARCH was an observational prospective cohort study conducted to assess the longitudinal association between alcohol consumption and biomarkers of microbial translocation and inflammation/altered coagulation [25]. The ARCH cohort included 351 participants recruited between November 2012 and June 2015 from clinical HIV and addiction care sites, non-clinical sites, and via snowball recruitment in St. Petersburg. Eligibility criteria for enrollment in the Russia ARCH study were: 1) age 18–70 years; 2) HIV infection; 3) having two contacts to enable follow-up; 4) residence within 100 km of St. Petersburg, Russia; 5) being ART-naïve (i.e., never having been on ART) and 6) having a telephone. Participants were excluded if they were not fluent in Russian or had a cognitive impairment that precluded them from providing informed consent. All participants provided informed consent in writing.

Participants were interviewed at baseline, 6, 12, 18, and 24 months post baseline. Of note, a subset (n = 254) of Russia ARCH participants participated in the ZINC trial, a double blind randomized placebo controlled trial of zinc supplementation among HIV-positive heavy drinkers. The present secondary data analysis was limited to Russia ARCH participants who completed a baseline assessment and were regular smokers (n = 309), defined as those who smoked at least one cigarette per day [26–28].

### Measures

Trained research staff conducted face-to-face study interviews, collecting data on demographics (age, sex), socioeconomic status (education level and individual income), food insecurity [29] (a nine-item Household Food Insecurity Access Scale), social support [30] (a five-item scale developed for the National Institute on Drug Abuse: Seek, Test, Treat and Retain Initiative [31]), years since first HIV positive test, HIV transmission risk factors, BMI, injection drug use in the past 30 days [32,33], and past 12-month alcohol dependence as measured by a score of 13 or more for women, and 15 or more for men on the AUDIT (Alcohol Use Disorders Identification test) [34].

### Independent variables

The main independent variable was a high level of depressive symptoms in the past 7 days. During the research assessment, participants self-administered the twenty-item CES-D.[21,22] We defined a high level of depressive symptoms as a CES-D score of ≥ 24, using a higher cutoff of the CES-D than the more commonly used cutoff of 16 [21]. We chose to use a higher cutoff, as false positive results on the order of 15% to 20% have resulted from use of a cut-off point of 16, leading some researchers to suggest that a higher cut-off point be used [35,36]. This is relevant because CES-D scores may be elevated in HIV-positive patients, as some symptoms of advanced HIV infection are similar to those of depression. We also performed sensitivity analyses using a CES-D cutoff of 16.

### Outcomes

The primary outcome was heavy smoking, defined as smoking > 20 cigarettes per day. While previous studies have defined heavy smoking as ≥ 20 cigarettes per day [37], our data only included the following categories of cigarettes per day: ≤10, 11–20, 21–30, and ≥ 31. A secondary outcome was a score of ≥ 5 on the six-item Fagerström Test for Nicotine Dependence, indicating moderate-very high dependence [23,24]. A score of 0–2 corresponds to very low nicotine dependence, 3–4 corresponds to low dependence, 5 corresponds to moderate dependence, 6–7 high dependence, and 8–10 very high dependence [38].

### Statistical analysis

We performed descriptive and multivariable regression analyses. In the descriptive analyses, we used chi-square test statistics to examine differences in proportions when analyzing categorical variables. We used two-sample *t* tests when analyzing continuous variables. Multivariable logistic regression analyses assessed associations between depressive symptoms and the outcomes (heavy smoking and moderate-very high nicotine dependence). The Hosmer-Lemeshow test was used to evaluate the goodness of fit of the logistic regression models. Based on knowledge from prior literature and clinical knowledge, we included the following potential covariates and confounders in the model: age, sex, education, income, running out of money for food/housing, injection drug use and alcohol dependence [39,40]. We did not expect zinc supplementation (received by some participants as part of the nested ZINC trial) to confound the relationships of interest; however, we assessed this in preliminary analyses. Among those enrolled in the ZINC trial, we compared outcomes for the current study by intervention arm and found no significant differences between randomized groups. As an exploratory analysis of effect modification, we included an interaction term between sex and depressive symptoms, given that men and women with depressive symptoms may have different smoking patterns. We evaluated possible multicollinearity by calculating Spearman correlation coefficients for each pair of independent variables and covariates prior to regression modeling. We verified that no pair of variables had a correlation greater than 0.40. Next, we calculated the variance inflation factor (VIF) for all terms in the regression models to further detect potential multicollinearity. We used a criterion of VIF>10 to indicate collinearity. The largest observed VIF was 1.09, suggesting multicollinearity was likely not an issue in the regression models. For hypothesis testing, we used two-tailed tests and a significance level of 0.05. Two-sided 95% confidence intervals were obtained for regression parameters of interest.

All analyses were performed using SAS 9.3 (Cary, North Carolina, USA). Institutional Review Boards of Boston University Medical Campus and First St. Petersburg Pavlov State Medical University approved the study protocol for the Russia ARCH study.

## Results

The analytic sample for the primary analysis was comprised of 309 regular smokers. Of these, 65 participants (21.0%) were heavy smokers (Table 1). The following variables appeared to differ between those with and without heavy smoking: sex, education level, running out of money for housing/food, physical component score, and injection drug use.

**Table 1:** Demographic and clinical characteristics of HIV-positive smokers^a^ in Russia according to smoking status.

Characteristic	No. (%)			
	Overall N = 309	Light-moderate Smoker N = 244	Heavy smoker N = 65	p-value^b^
Sex				0.033
Male	219 (70.9)	166 (75.8)	53 (24.2)	
Age, y, mean (sd)	33.5 (5.3)	33.5 (5.4)	33.7 (4.7)	0.793
Education				0.010
< 9 grades	11 (3.6)	7 (63.6)	4 (36.4)	
9 grades	52 (16.8)	35 (67.3)	17 (32.7)	
11 grades/ specialized secondary education/college	198 (64.1)	167 (84.3)	31 (15.7)	
Incomplete higher education	32 (10.4)	21 (65.6)	11 (34.4)	
Higher education	16 (5.2)	14 (87.5)	2 (12.5)	
Individual Income				0.880
Low (0–25,000 Rubles)^c^	221 (71.5)	175 (79.2)	46 (20.8)	
Marital status				0.284
Married/long-term partner	161 (52.1)	123 (76.4)	38 (23.6)	
Separate/divorced/widowed	64 (20.7)	55 (85.9)	9 (14.1)	
Never married	84 (27.2)	66 (78.6)	18 (21.4)	
Ran out of money for housing/food, past 12 months				0.050
Yes	186 (60.2)	140 (75.3)	46 (24.7)	
Food Insecure				0.139
Yes	165 (53.4)	125 (75.8)	40 (24.2)	
AUDIT: Alcohol Dependence				0.134
Yes	182 (58.9)	149 (81.9)	33 (18.1)	
Past 30 day injection drug use				0.014
Yes	130 (42.1)	94 (72.3)	36 (27.7)	
Social support, mean (sd)	20 (5)	20 (5)	20 (5)	0.782
Nicotine Dependence^d^ on Fagerstrom				<0.001
Low-moderate	118 (38.2)	113 (95.8)	5 (4.2)	
Moderate-very high	191 (61.8)	131 (68.6)	60 (31.4)	
Years since first HIV positive test, mean (sd)	7.2 (4.8)	7.1 (4.9)	7.7 (4.5)	0.332
BMI, mean (sd)	22.8 (3.1)	22.7 (3.1)	23.1 (3.0)	0.354
HIV transmission risk				0.687
Men who have sex with men/IDU	4 (1.3)	3 (75.0)	1 (25.0)	
Men who have sex with men	3 (1.0)	2 (66.7)	1 (33.3)	
IDU	261 (84.5)	203 (77.8)	58 (22.2)	
Presumed heterosexual/blood/blood products	4 (1.3)	4 (100)	0	
Presumed heterosexual only	35 (11.3)	30 (85.7)	5 (14.3)	
Refused or none selected	2 (0.6)	2 (100)	0	
Log_10_ HIV viral load, mean (sd) (N = 306)	9.7 (2.6)	9.7 (2.5)	9.9 (2.8)	0.583
HIV Symptom Count ^e^				0.24
High	137 (44.3)	104 (75.9)	33 (24.1)	
Physical Component Score, mean (sd)^f^	49.1 (8.1)	49.6 (7.7)	47.3 (9.4)	0.041
Depression: CES-D Score 24+^g^	79 (25.6)	58 (73.4)	21 (26.6)	0.161
No Depression	230 (74.4)	186 (80.9)	44 (19.1)	

^a^ Participants who smoked at least seven cigarettes per week

^b^ We used chi-square test statistics when analyzing categorical variables, and two-sample *t* tests when analyzing continuous variables.

^c^ 25,000 rubles ranged from the equivalent of approximately US $799 when ARCH study recruitment began in November 2012 to US $468 in June 2015 when ARCH recruitment was completed

^d^ Moderate-very high dependence on the Fagerstrom test for nicotine dependence defined as a score ≥ 5

^e^ Dichotomized at the sample’s mean as high vs low

^f^ Assessed using Veterans Rand 12-Item Health Survey (VR-12); higher scores indicative of better health

^g^ High level of depression symptoms in the past 7 days defined as a CES-D score of ≥ 24

Twenty-seven percent of participants with depressive symptoms were heavy smokers (n = 21), relative to one-fifth of participants without high levels of depressive symptoms (n = 44). Seventy percent of participants with high levels of depressive symptoms had moderate-very high nicotine dependence (n = 55), relative to 59% of participants without high levels of depressive symptoms (n = 136). In multivariable logistic regression analyses, the presence of high levels of depressive symptoms was not significantly associated with either heavy smoking (adjusted odds ratio [aOR] 1.50, 95% CI 0.78–2.89; Table 2) or with moderate-very high levels of nicotine dependence (aOR 1.35, 95% CI 0.75–2.41; Table 3). In terms of covariates, female sex, higher levels of education, and alcohol dependence appeared to be associated with a lower odds of heavy smoking, while injection drug use in the past 30 days and running out of money for housing/food appeared to be associated with an increased odds of heavy smoking. In these models, the interaction term between sex and depression was not significant. In sensitivity analyses using a CES-D cutoff of 16, these findings did not change for models examining heavy smoking (S1 Appendix Table, Associated factors of heavy smoking among HIV-positive smokers in Russia.). However, the presence of high levels of depressive symptoms, based on the lower CES-D cutoff, was significantly associated with moderate-very high levels of nicotine dependence (aOR 1.71, 95% CI 1.04–2.80; S2 Appendix Table, Associated factors of moderate-very high levels of nicotine dependence among HIV-positive smokers in Russia).

**Table 2:** Associated factors of heavy smoking^a^ among HIV-positive smokers^b^ in Russia.

	Unadjusted Odds Ratio (95%CI)	p-value	Adjusted Odds Ratio^c^ (95%CI)	p-value
High depressive symptoms, CES-D ≥ 24^d^	1.53 (0.84, 2.78)	0.163	1.50 (0.78, 2.89)	0.223
CES-D < 24	1.00		1.00	
Sex		0.036		0.014
Female	0.48 (0.24, 0.95)		0.39 (0.19, 0.83)	
Male	1.00		1.00	
Education		0.008		0.019
≥ 9 grades	0.44 (0.24, 0.81)		0.45 (0.23, 0.88)	
< 9 grades	1.00		1.00	
Individual income		0.880		0.771
> 25,000 rubles^e^	1.05 (0.57, 1.91)		1.10 (0.57, 2.15)	
≤ 25,000 rubles	1.00		1.00	
Past 30 day injection drug use (IDU)	1.98 (1.14, 3.44)	0.015	1.90 (1.06, 3.41)	0.032
No IDU in past 30 days	1.00		1.00	
Alcohol Dependence on AUDIT	0.66 (0.38, 1.14)	0.135	0.52 (0.29, 0.94)	0.030
No alcohol dependence	1.00		1.00	
Ran out of money for housing/food	1.80 (1.00, 3.25)	0.052	1.94 (1.03, 3.64)	0.040
Did not run out of money for housing/food	1.00		1.00	
Age (per 5 year increase)	1.04 (0.80, 1.34)	0.792	1.01 (0.75, 1.35)	0.957

^a^ Heavy smoking defined as > 20 cigarettes per day

^b^ Participants who smoked at least seven cigarettes per week

^c^ Adjusted for sex, education, income, running out of money for housing/food, injection drug use, AUDIT score, and age. Hosmer-Lemeshow Goodness-of-Fit test Chi-square 6.61, 8 degrees of freedom, p = 0.57

^d^ High level of depression symptoms in the past 7 days defined as a CES-D score of ≥ 24

^e^ 25,000 rubles ranged from the equivalent of approximately US $799 when ARCH study recruitment began in November 2012 to US $468 in June 2015 when ARCH recruitment was completed

**Table 3:** Associated factors of moderate-very high levels of nicotine dependence^a^ among HIV-positive smokers^b^ in Russia.

	Unadjusted Odds Ratio (95%CI)	P value	Adjusted Odds Ratio (95%CI)^c^	P value
High depressive symptoms, CES-D ≥ 24^d^	1.58 (0.92, 2.74)	0.099	1.35 (0.75, 2.41)	0.318
CES-D < 24	1.00		1.00	
Sex		0.233		0.063
Female	0.74 (0.45, 1.22)		0.59 (0.34, 1.03)	
Male	1.00		1.00	
Education		0.081		0.183
≥ 9 grades	0.58 (0.32, 1.07)		0.65 (0.35, 1.23)	
< 9 grades	1.00		1.00	
Individual income		0.163		0.448
> 25,000 rubles^e^	0.70 (0.42, 1.16)		0.81 (0.47, 1.40)	
≤ 25,000 rubles	1.00		1.00	
Past 30 day injection drug use (IDU)	1.46 (0.91, 2.34)	0.116	1.29 (0.79, 2.13)	0.309
No IDU in past 30 days	1.00		1.00	
Alcohol Dependence on AUDIT	1.36 (0.86, 2.17)	0.191	1.16 (0.71, 1.90)	0.563
No alcohol dependence	1.00		1.00	
Ran out of money for housing/food	2.23 (1.39, 3.57)	<0.001	2.19 (1.34, 3.58)	0.002
Did not run out of money for housing/food	1.00		1.00	
Age (per 5 year increase)	0.91 (0.73, 1.13)	0.375	0.87 (0.69, 1.10)	0.256

^a^ Moderate-very high dependence on the Fagerstrom test for nicotine dependence defined as a score ≥ 5

^b^ Participants who smoked at least seven cigarettes per week

^c^ Adjusted for sex, education, income, running out of money for housing/food, injection drug use, AUDIT score, and age. Hosmer-Lemeshow Goodness-of-Fit test Chi-square 6.450, 8 degrees of freedom, p = 0.709.

^d^ High level of depression symptoms in the past 7 days defined as a CES-D score of ≥ 24

^e^ 25,000 rubles ranged from the equivalent of approximately US $799 when ARCH study recruitment began in November 2012 to US $468 in June 2015 when ARCH recruitment was completed

## Discussion

Among HIV-positive smokers enrolled in the Russia ARCH study, we were unable to detect an association between depressive symptoms and heavy smoking or moderate-very high nicotine dependence. Our findings are consistent with a US study by Webb et al., which found that among a clinic-based sample of HIV-positive patients, depression was not associated with heavy smoking [18]. However, others in the US have found that depression is associated with higher nicotine dependence and smoking prevalence among HIV-positive individuals [17,41].

It is possible that social norms regarding smoking in Russia could explain our findings. Social norms in Russia support ongoing tobacco use; smoking cigarettes is the norm, and quitting has not become normative. Smoking remains largely socially accepted, providing little extrinsic motivation for persons with or without depressive symptoms to quit smoking. Tobacco control policies in Russia may also have affected our findings. Russia has begun to implement key mandates of the WHO Framework Convention on Tobacco Control [42], such as regular monitoring of tobacco use and tobacco prevention efforts, public smoking bans, mass-media campaigns about the dangers of tobacco use, and bans on tobacco advertising [43]. However, few of these measures have targeted HIV-positive populations, who have high rates of comorbid substance use disorders.

The prevalence of substance use disorders was high among our study population, with over half of all participants having alcohol dependence or recent injection drug use. Individuals with mental disorders such as depression may use alcohol and/or drugs as a form of self-medication.[44] Thus, it is possible that we did not observe the expected association between depression and heavy smoking because participants were self-medicating their depression with alcohol and/or drug use. In addition, as in many addiction treatment systems globally, there is little attention paid to addressing tobacco use in Russia’s Narcology (i.e., addiction care) system when substance use disorders are the primary concern.[43] Thus, heavy smoking may not be limited to those individuals with depression among persons with addictions.

In addition, the price of cigarettes remains low in Russia. Ross et al have attributed the low cigarette prices to insufficient tobacco tax policies [45]. Tobacco taxes in Russia are far below the 67 to 80% rate recommended by the World Bank to reduce tobacco use [45]. This again provides little disincentive for cigarette purchasing to smokers, with or without mental health problems.

Robust tobacco control measures in the US have reduced smoking rates to an extent that remaining US smokers tend to be those with vulnerabilities that may hinder quitting, such as lower socioeconomic status, mental health conditions, or substance use disorders [46]. The apparent lack of association between depressive symptoms and heavy smoking in this Russian cohort suggests that in order to decrease the tobacco burden among HIV-positive smokers, evidence-based tobacco control strategies need to be targeted to all HIV-positive smokers, regardless of comorbid depression. Given that people receiving antiretroviral therapy living with HIV who smoke lose more years of life expectancy to their tobacco use than to their HIV infection, tobacco control measures should target this population [47]. Such measures include tobacco taxation [48] and cessation support. Finally, tobacco control measures might also target men and smokers with low socioeconomic status, as we observed that these groups were more likely to be heavy smokers and to have higher levels of nicotine dependence.

Our study has several limitations. First, our analyses may have been underpowered to detect an association between depressive symptoms and heavy smoking. In *post-hoc* power calculations, based on the assumption that 19% of the participants without depressive symptoms reported heavy smoking (observed at study entry), our study would have approximately 80% power to detect an odds ratio as small as 2.4. Thus, it is likely that the study did not have high power to detect the observed magnitude of association between depressive symptoms and heavy smoking. We also did not have data on biological differences in the rate of nicotine metabolism among participants; participants who were fast metabolizers of nicotine may have smoked more heavily [49].

In conclusion, we were unable to detect an association between depressive symptoms and heavy smoking or moderate-very high nicotine dependence in this cohort of HIV-positive Russian smokers. Future research investigating these associations in larger population samples is warranted. Among people with HIV and substance use, who have high smoking prevalence overall, and a sizable minority of whom are heavy smokers with high levels of nicotine dependence, health policies that implement a broad anti-smoking approach are warranted irrespective of depressive symptoms.

## Supporting information

S1 Appendix Table Associated factors of heavy smoking among HIV-positive smokers in Russia. (DOCX)

S2 Appendix Table Associated factors of moderate-very high levels of nicotine dependence among HIV-positive smokers in Russia. (DOCX)
