# Supplementary material for: Depression and smoking characteristics among HIV-positive smokers in Russia: A cross-sectional study
Source: PLoS One. 2018 Feb 6;13(2):e0189207. doi: 10.1371/journal.pone.0189207 (PMC5800551; doi:10.1371/journal.pone.0189207)
Supplement: S1 Appendix Table — (DOCX) [file pone.0189207.s001.docx]

**S1 Appendix Table. Associated factors of heavy smoking^a^ among HIV-positive smokers^b^ in Russia**

|  | **Unadjusted Odds Ratio**  **(95%CI)** | **p-value** | **Adjusted Odds Ratio^c^**  **(95%CI)** | **p-value** |
| --- | --- | --- | --- | --- |
| High depressive symptoms, CES-D > 16  CES-D < 16 | 1.44 (0.83, 2.49)  1.00 | 0.195 | 1.22 (0.67, 2.21)  1.00 | 0.510 |
| Sex  Female  Male | 0.48 (0.24, 0.95)  1.00 | 0.036 | 0.40 (0.19, 0.85)  1.00 | 0.017 |
| Education  > 9 grades  < 9 grades | 0.44 (0.24, 0.81)  1.00 | 0.008 | 0.45 (0.23, 0.87)  1.00 | 0.017 |
| Individual income  > 25,000 rubles^d^  ≤ 25,000 rubles | 1.05 (0.57, 1.91)  1.00 | 0.880 | 1.05 (0.55, 2.03)  1.00 | 0.876 |
| Past 30 day injection drug use (IDU)  No IDU in past 30 days | 1.98 (1.14, 3.44)  1.00 | 0.015 | 1.93 (1.08, 3.46)  1.00 | 0.027 |
| Alcohol Dependence on AUDIT  No alcohol dependence | 0.66 (0.38, 1.14)  1.00 | 0.135 | 0.54 (0.30, 0.97)  1.00 | 0.038 |
| Ran out of money for housing/food  Did not run out of money for housing/food | 1.80 (1.00, 3.25)  1.00 | 0.052 | 1.90 (1.01, 3.60)  1.00 | 0.048 |
| Age (per 5 year increase) | 1.04 (0.80, 1.34) | 0.792 | 1.02 (0.76, 1.37) | 0.885 |

^a^ Heavy smoking defined as > 20 cigarettes per day

^b^ Participants who smoked at least seven cigarettes per week

^c^ Adjusted for sex, education, income, running out of money for housing/food, injection drug use, AUDIT score, and age. Hosmer-Lemeshow Goodness-of-Fit test Chi-square 9.37, 8 degrees of freedom, p=0.312

^d^ 25,000 rubles ranged from the equivalent of approximately US $799 when ARCH study recruitment began in November 2012 to US $468 in June 2015 when ARCH recruitment was completed
