# Supplementary material for: Depression and smoking characteristics among HIV-positive smokers in Russia: A cross-sectional study
Source: PLoS One. 2018 Feb 6;13(2):e0189207. doi: 10.1371/journal.pone.0189207 (PMC5800551; doi:10.1371/journal.pone.0189207)
Supplement: S2 Appendix Table — (DOCX) [file pone.0189207.s002.docx]

**S2 Appendix Table. Associated factors of moderate-very high levels of nicotine dependence^a^ among HIV-positive smokers^b^ in Russia**

|  | **Unadjusted Odds Ratio**  **(95%CI)** | **P value** | **Adjusted Odds Ratio^c^**  **(95%CI)** | **P value** |
| --- | --- | --- | --- | --- |
| High depressive symptoms, CES-D > 16  CES-D < 16 | 2.05 (1.28, 3.28)  1.00 | 0.003 | 1.71 (1.04, 2.80)  1.00 | 0.035 |
| Sex  Female  Male | 0.74 (0.45, 1.22)  1.00 | 0.233 | 0.58 (0.34, 1.02)  1.00 | 0.057 |
| Education  > 9 grades  < 9 grades | 0.58 (0.32, 1.07)  1.00 | 0.081 | 0.66 (0.35, 1.26)  1.00 | 0.209 |
| Individual income  > 25,000 rubles^d^  ≤ 25,000 rubles | 0.70 (0.42, 1.16)  1.00 | 0.163 | 0.81 (0.47, 1.40)  1.00 | 0.461 |
| Past 30 day injection drug use (IDU)  No IDU in past 30 days | 1.46 (0.91, 2.34)  1.00 | 0.116 | 1.27 (0.77, 2.08)  1.00 | 0.352 |
| Alcohol Dependence on AUDIT  No alcohol dependence | 1.36 (0.86, 2.17)  1.00 | 0.191 | 1.17 (0.71, 1.92)  1.00 | 0.527 |
| Ran out of money for housing/food  Did not run out of money for housing/food | 2.23 (1.39, 3.57)  1.00 | <0.001 | 2.02 (1.23, 3.34)  1.00 | 0.006 |
| Age (per 5 year increase) | 0.91 (0.73, 1.13) | 0.375 | 0.88 (0.97, 1.11) | 0.299 |

^a^ Moderate-very high dependence on the Fagerstrom test for nicotine dependence defined as a score > 5

^b^ Participants who smoked at least seven cigarettes per week

^c^ Adjusted for sex, education, income, running out of money for housing/food, injection drug use, AUDIT score, and age. Hosmer-Lemeshow Goodness-of-Fit test Chi-square 12.984, 8 degrees of freedom, p=0.112.

^d^ 25,000 rubles ranged from the equivalent of approximately US $799 when ARCH study recruitment began in November 2012 to US $468 in June 2015 when ARCH recruitment was completed
